# Universal School Meals in the US: What Can We Learn from the Community Eligibility Provision?

**DOI:** 10.3390/nu13082634

**Published:** 2021-07-30

**Authors:** Tatiana Andreyeva, Xiaohan Sun

**Affiliations:** Rudd Center for Food Policy and Obesity, Department of Agricultural and Resource Economics, University of Connecticut, 1 Constitution Plaza, Hartford, CT 06103, USA; xiaohan.sun@uconn.edu

**Keywords:** child nutrition, universal school meals, schools

## Abstract

Changes in school meal programs can affect well-being of millions of American children. Since 2014, high-poverty schools and districts nationwide had an option to provide universal free meals (UFM) through the Community Eligibility Provision (CEP). The COVID-19 pandemic expanded UFM to all schools in 2020–2022. Using nationally representative data from the Early Childhood Longitudinal Study: Kindergarten Class of 2010–2011, we measured CEP effects on school meal participation, attendance, academic achievement, children’s body weight, and household food security. To provide plausibly causal estimates, we leveraged the exogenous variation in the timing of CEP implementation across states and estimated a difference-in-difference model with child random effects, school and year fixed effects. On average, CEP participation increased the probability of children’s eating free school lunch by 9.3% and daily school attendance by 0.24 percentage points (*p* < 0.01). We find no evidence that, overall, CEP affected body weight, test scores and household food security among elementary schoolchildren. However, CEP benefited children in low-income families by decreasing the probability of being overweight by 3.1% (*p* < 0.05) and improving reading scores of Hispanic children by 0.055 standard deviations. UFM expansion can particularly benefit at-risk children and help improve equity in educational and health outcomes.

## 1. Introduction

On a typical school day, almost 30 million students eat lunch served through the National School Lunch Program (NSLP) and 14.8 million students have breakfast via the School Breakfast Program [1]. Most of these meals—85% for breakfast, 74% for lunch [1]—are served free or at reduced prices, targeting children at greater risk for food insecurity and poor diet. All school meals must meet USDA nutrition standards [2] that ensure all children’s access to nutritious foods. Participation in the NSLP has been linked to improvements in children‘s dietary intake and health, with most significant gains observed among nutritionally-disadvantaged low-income children [3,4,5]. As school meals are almost universal (99% of US public schools and 83% of private schools participate in NSLP [6]), any changes in school nutrition have potential to affect the diets of millions of American children. Increasing evidence suggests that the quality of diet can impact academic achievement, school attendance, long-life income, and health [7,8], so changes in school nutrition have implications far beyond dietary outcomes. 

In 2010, the Healthy, Hunger-Free Kids Act (HHFKA) initiated a number of changes in the school meal programs, including the establishment of the Community Eligibility Provision (CEP) that was intended to increase school meal participation and improve food security among children in high-poverty communities. Despite widespread access to the NSLP, many eligible low-income students (from 16% in elementary schools to 36% in high schools) did not participate due to common stigmatization, access to competitive foods and other barriers [9,10]. CEP allowed the provision of universal free meals (UFM) in high-poverty schools if ≥40 percent of students in the prior year could be directly certified for free meals (i.e., students identified as participating in other food assistance programs, such as the Supplemental Food Assistance Program). CEP eliminated the household application process and streamlined meal counts and claiming procedures, which was expected to reduce the administrative burden and related costs to local school districts [11,12]. On a broader scale, the idea behind CEP was that better nutrition through CEP for children previously not benefiting from school meals could improve child’s wellbeing and reduce socio-economic disparities in education and health.

Important to the identification strategy in this study, CEP was implemented gradually over four years, with first school districts becoming eligible for CEP in school year (SY) 2011–2012 in three pilot states (IL, KY, MI), followed by districts in four states (DC, NY, OH, WV) next year, and additional four states (GA, FL, MA, MD) in SY 2013–2014. All remaining states became eligible in SY 2014–2015 [12]. In its second year of national CEP availability, more than 18,000 high-poverty schools, or half of eligible schools, chose to participate in CEP [13]. School districts of a bigger size, located in the Southeast, urban districts, and with at least one school receiving full reimbursements were more likely to adopt the CEP [14]. As of 2020, 33,171 schools across the nation, or 68% of eligible schools, adopted CEP [15], which significantly expanded access to free nutritious school meals. 

Recent evaluations, most from specific states, have found that the adoption of CEP has improved school meal participation [16,17,18,19,20,21], attendance [18,22,23,24], academic achievement [18,19,23,24], and school food service finances [25,26]. At the same time, CEP was shown to reduce out-of-school suspension and expulsion rates [23,27]. Research on obesity implications has produced mixed results, varying from a reduction in body mass index (BMI) across grades [28], increased BMI percentile [29], and reduced BMI and obesity among select student groups only [18,26,29]. It is yet unknown whether CEP implementation has translated into measurable nutrition and health gains for children nationwide, and how these gains were distributed across population sub-groups. Potential co-benefits of improved school meal participation need further documentation, including effects on household food security, academic achievement, family and school finances. 

This study provides a comprehensive, nationwide assessment of the multiple impacts that CEP has brought to low-income communities in its early years (2011–2016), including effects on school meal participation, attendance, academic achievement, children’s body weight, and household food security. Drawing on the exogenous variation in the timing of CEP adoption across states, we used a quasi-experimental approach of comparing the temporal change in outcomes between the treatment and control groups (i.e., a difference-in-difference model) to provide plausibly causal estimates of the CEP effects in a national longitudinal sample of elementary schoolchildren. To understand how vulnerable groups were affected across the spectrum of outcomes, the study included multiple sub-group analyses. 

## 2. Materials and Methods

### 2.1. Data

We used a nationally representative panel of U.S. children from the Early Childhood Longitudinal Study, Kindergarten Class of 2010–2011 (ECLS-K:2011), which selected children from public and private schools attending full- or part-day kindergarten in 2010–2011. ECLS-K:2011 focused on children’s early school experiences, beginning with kindergarten entry and through the fifth grade, with data collection in the fall and spring of kindergarten, first grade and second grade, and the spring of third, fourth and fifth grades. The study included parent, teacher, school administrator, and child-level data on children’s cognitive and social development, home environment, test scores, child’s health, body weight and height, and behavior [30].

We used child assessment, parent-, teacher- and school administrator-reported data from all six spring waves. To enable linkages between the school’s location and child-level outcomes, we used a restricted version of the ECLS-K:2011 with geographic identifiers, which was provided by the National Center for Education Statistics at the US Department of Education [30]. The sample had children from 41 states for the period of 2011–2016, including 9 CEP pilot states (FL, GA, IL, MA, MD, MI, NY, OH and WV). 

Data on school-level CEP eligibility and participation in all states in SY 2014–2015 and SY 2015–2016 were obtained from the Food Research and Action Center (FRAC) and the Center on Budget and Policy Priorities (CBPP) [31,32]. To define CEP eligibility and participation for pilot states during SY 2011–2014, we collected administrative data from State Departments of Education and another researcher studying CEP [19]. 

As the ECLS-K:2011 did not include school names and did not collect data on CEP, additional steps were necessary to enable a link between the ECLS-K:2011 with administrative data on school CEP eligibility and participation. School names were collected from external data sources, including the Common Core of Data (CCD) for public schools and the Private School Universe Survey (PSS) for private schools [33,34]. The ECLS-K:2011 included CCD and PSS identifiers, which were used to locate each school by name and location to enable a merge with administrative data on CEP. The merge used a fuzzy matching function in STATA based on school, school district, and state names. All matching results were manually checked. 

### 2.2. Sample Selection

The study population was elementary schoolchildren (K through 5th grade) that attended kindergarten in 2010–2011. As CEP eligible schools and/or school districts were not required to participate in CEP, participation decisions were choices that could be correlated with unobservable factors also affecting school and child-level outcomes. Reverse causality is another threat in that a school/district’s performance on certain outcomes could affect its decision to participate in CEP. To demonstrate the point on potential selection bias into CEP, Table 1 demonstrates significant differences in the observable mean characteristics of schools that chose to participate in CEP and non-participating schools, including those that were eligible but selected not to participate. To account for potential selection bias and consistent with previous economic research [18,19], the analytic sample in this study was limited to children attending schools that participated in CEP at any point in our data in 2011–2016, excluding children in CEP ineligible (i.e., higher income) schools/districts and CEP eligible schools/districts that did not participate in CEP within two years of its nationwide implementation. 

### 2.3. Measures

The study included multiple outcomes of the CEP effects, including school meal participation, school attendance, academic achievement, child’s body weight, and household food security. School meal participation was assessed based on two measures: (1) school administrators’ reported percentage of children eligible for free or reduced-price lunch—referred below as free school meals, and (2) parental reports of their child’s receiving free school lunch. Attendance was reported by school administrators as a percent of enrolled children attending school on an average day. Academic achievement included assessments of a student’s proficiency in reading, math and science that were measured by direct child cognitive assessment scale scores using the Item Response Theory (IRT) procedures. The test performance metrics were transformed into z-scores normalized using grade-by-year means and variances of each subject.

The child’s height and weight, which were measured by the ECLS-K:2011 study’s personnel, were used to construct age- and gender-specific BMI percentiles and z-scores. Indicators for child overweight (BMI percentile: 85.0–94.99) and overweight/obesity (BMI percentile: 85.0 and above) were used to assess children’s BMI status. Finally, household food security was assessed based on parental responses to the 10-item version of the USDA’s adult food security measure. Three binary outcomes were created, including an indicator of a food secure household (<3 affirmative responses to 10 food security items), one for low food security (3–5 affirmative responses), and one for very low food security (≥6 affirmative responses). Adult food security was used as a measure of household food security given few reports of food disruptions among children and a very small sample size for child food security.

The main independent variable was an indicator for whether a child attended a CEP-participating school during the academic year. Socio-demographic controls at the child and household-level included the child’s gender, race/ethnicity, household income below the 200% federal poverty level, primary language at home other than English, and mother’s education. Child’s birth weight was used as a covariate in BMI models only. At the school level, percentage of poverty in a school district and the duration of CEP participation were added as covariates.

### 2.4. Estimation Model

The identification strategy was based on the variation in the timing of state CEP eligibility, which was set independently of school districts by the US Department of Agriculture [12]. The different timing of CEP adoption allowed the use of a difference-in-difference model to identify causality. Assuming random nature of the USDA’s selection of pilot states and that early implementation upon eligibility had no more benefits than later implementation, the difference-in-difference model can measure the causal effect of CEP by comparing early to late CEP-adopting schools. The model was specified as:

(1) $$Y_{jt}=\beta_{0}+\beta_{1}CEP_{jt}+\beta_{2}X_{jt}+\mu_{s}+\gamma_{t}+\pi_{j}+\varepsilon_{jt},$$

where $Y_{jt\;}$ is the outcome for child *j* in year *t*, $CEP_{jt}$ is a dichotomous measure of whether a child’s school participated in CEP in year *t*, $X_{jt}$ is a vector of child, family, and school-level covariates for child *j* in year *t*, $\mu_{s}$ is a school fixed effect for time-invariant school characteristics, $\gamma_{t}$ is a year fixed effect, $\pi_{j}$ represents a child random effect to control for time-invariant heterogeneity at the child level, and $\varepsilon_{jt}$ is the error term. $\beta_{1}$ captures a causal estimate of the CEP effects under the assumption that the CEP adoption time is exogenous. This means that both the school’s adoption time and the adoption time within students was random. A Hausman test was conducted to test the validity of the child random-effects model by comparing the child random-effects and fixed-effects models, with results favoring the random-effects model. Robust standard errors were clustered at the child level. All models were estimated using a linear probability model based on the distribution of residuals that were almost equally and randomly distributed centering around zero for all outcomes. 

As CEP launched as a pilot in three states in school year (SY) 2011–2012, $CEP_{jt}$ was set to 0 for all children in school year 2010–2011 (kindergarten). The duration of the pre- and post-CEP period varied depending on the child’s state and the timing of CEP adoption. Children residing in pilot states that implemented CEP earlier than the rest of the country had a longer exposure to CEP, beginning in the 1st to 5th grade. Children residing in the states that adopted CEP in SY 2014–2015 had up to two years of the program exposure, in 4th and 5th grades only. We also tested the difference between CEP early adopters (adoption before 2014–2015) and late adopters (2015–2016). 

The key assumption of the difference-in-difference model is the parallel trends, which implies that the trends of change in the control group would be the same as the trends of change in the treatment group in the absence of the policy [35]. For example, if outcomes in the treatment group were improving faster than in the control group before the policy change, the difference-in-difference model would overestimate the policy effect. This study tested the parallel trends assumption using an event study framework to account for the variation in the timing of the policy change (i.e., CEP eligibility), which is consistent with prior economic research on CEP [18,19]. Specifically, the model in equation (1) was re-estimated by including a set of indicators to capture the years before and after the CEP introduction. The leads and lags of the CEP indicators included one year, two years and three or more years prior to the CEP adoption, and similar periods following the policy change. The results in Supplementary Materials Figures S1–S3 for the full sample show that the coefficients in the years prior to the CEP adoption were not statistically different from zero, supporting the parallel trends assumption.

#### Sensitivity Analyses and Robustness Checks

We conducted a variety of tests to check robustness of our results. One sensitivity analysis tested the hypothesis that children could switch schools to get access to UFM, which would have violated our model’s assumption that the adoption time within students was exogenous and unrelated to unobserved characteristics of the children. To test this hypothesis, we limited the sample to students whose change in the CEP status was due to their school’s change rather than students switching schools. Specifically, 42% of children in our analytic sample changed their CEP participation status by changing schools—due to moving, a school switch, or the school district design of separate K-2 and 3–5 schools. At the same time, 58% students had the change because their school implemented CEP.

Additional sensitivity analyses included estimation of generalized linear models, alternative outcomes (e.g., BMI percentile instead of BMI z-score), estimating a Beta regression for attendance outcomes, using two datapoints DID model by grade, comparing regression with or without panel weights, and school covariates instead of school fixed effects (which did not pass the Hausman test). We further tested sensitivity of our results by re-estimating model (1) using child fixed effects instead of child random effects. Results for the child fixed effects models were similar with the main results presented. Additional sensitivity analysis included the exclusion of children with extreme values on BMI z-scores. 

To assess heterogeneity in the effects of CEP participation across children, the study included several subgroup analyses: i.e., by household income, child’s race/ethnicity, prior receipt of free/reduced price school meals, and language at home other than English. 

## 3. Results

The analytic sample of children in CEP-participating schools included around 2500 children (all sample size numbers were rounded to the nearest 50 per the ECLS-K:2011 disclosure requirements). Table 2 presents descriptive statistics for the study outcomes and covariates for children attending elementary schools that participated in CEP at any point during 2011–2016 (our analytic sample) and children from schools not on CEP during this period. Results show significant disparities in the characteristics of children based on their school CEP participation status, including higher rates of economically-disadvantaged children, racial/ethnic minorities, children speaking non-English at home, and children attending high-poverty, urban schools in the CEP group. Further, children in elementary schools that adopted CEP by 2015–2016 were more likely to have lower academic achievement, participate in school meal programs, and have higher rates of obesity and household food insecurity than their peers in non-participating schools. These large differences highlight the importance of restricting the analytic sample in this CEP study to children in CEP schools only. 

Results from the difference-in-difference model of the CEP effects for U.S. elementary schoolchildren in 2011–2016 are presented in Table 3. The full sample results are shown in Column 1, suggesting a statistically significant increase in school meal participation and receipt of free school lunch, as well as improved school attendance, but no measurable changes in other outcomes. Specifically, CEP increased access to free school lunch by 2.4 percentage points (*p* < 0.01). Based on parental reports, CEP increased the probability of children getting free school lunch by 9.3% (*p* < 0.01). Furthermore, CEP also improved school attendance by increasing the percent of students attending daily by 0.24 percentage points (*p* < 0.01). Models for academic achievement outcomes did not pass the parallel trend assumption and are therefore not reported, but no significant results were identified in the full analytic sample. CEP was found to have no effect on body weight outcomes and household food security among elementary schoolchildren in 2011–2016. 

Sub-group analyses revealed important heterogeneity in the CEP effects across children of diverse backgrounds, with several results indicating a larger and more significant effect on children from less-advantaged families. For example, children from low-income families (Column 2) that could be affected by UFM if not previously participating in the free school meal programs, had a reduction in the probability of being overweight by 3.1% (*p* < 0.05). This result was in addition to improved school meal participation and school attendance also seen in the full sample, with estimated effects of the equivalent magnitude. The sub-sample of Hispanic children (Column 3) showed a marginally significant CEP-attributable increase in reading scores, on average by 0.055 standard deviations (*p* < 0.1). Math and science scores, however, were not affected, while all models of academic achievement for Hispanic children passed the parallel trend test. Furthermore, among children not receiving free/reduced price lunch before CEP, we saw a particularly large increase in the probability of a child’s receiving free school meals, by 40.7% (*p* < 0.01), but other outcomes were not significant. Finally, the only significant result for children whose primary language at home was not English was increased school attendance, by 0.436 percentage points (*p* < 0.05). For all sub-groups, there was no evidence that CEP affected household food security in a significant way. Our sensitivity analyses showed results that were consistent with the results of the full sample or the main specification model.

## 4. Discussion

This study finds that the adoption of CEP and UFM in the early years of the program’s life has increased school meal participation and students’ receipt of free school meals, improved school attendance, and had additional academic and health benefits for several sub-groups of at-risk children. Hispanic children were shown to particularly gain from CEP by having higher reading scores, while children from low-income families had a lower risk of being overweight with the adoption of CEP in their schools. We find no evidence that CEP affected household food security. Results are robust across multiple model specifications in our national sample of elementary schoolchildren in 2011–2016. 

The findings of improved participation in school meals and greater use of free meals are consistent with data from prior studies, including two recent systematic reviews on the effects of CEP and UFM [36,37]. The magnitude of results in our national sample is, however, more modest, potentially due to our study design or the sample of younger elementary schoolchildren. Our research and prior studies confirm that CEP achieves its primary goal of increasing school meal participation and expanding reach for the School Breakfast Program and the National School Lunch Program [17,18,19,20,21,23,36,37]. We find evidence for improvements in school meal participation based on data from two independent sources, including school administrators and parents, which makes the findings more reliable. It is encouraging that the improvements seen in the school meal reach are particularly large for children who did not receive free/reduced-price school meals prior to the CEP adoption in their schools. It is possible these children were eligible, but did not participate due to stigma, lack of knowledge or administrative burdens [10,38]. CEP has removed these barriers for children and their families.

The observed improvement in school attendance is modest at 0.24 percentage points in the percent of children attending school, which fits mixed results on this outcome in the literature. Results vary from lower absences among elementary schoolchildren, but not middle-schoolers [24], to reduced poor attendance only among economically disadvantaged children in the second (but not first) year of the CEP implementation [22], and increased absences across grades [23]. School absenteeism is an important predictor of student achievement, high school and college graduation rates, adult income, and health [39,40]. Even modest improvements in attendance with CEP render important supports to the program’s wider impacts outside of the direct effect on school meal participation. Future studies should continue assessments of the CEP effects on attendance and related long-term outcomes. 

Our results on academic achievement are distinct from previous state-specific studies. It is possible that high heterogeneity in our sample of 41 states can explain the model’s failure to confirm the parallel trend assumption for test scores, except for results among Hispanic children that are likely to be residing more narrowly in the Western and Southwestern states. Our sample size was too small to conduct state- or region-specific analyses. We tried an alternative model using school covariates and state covariates for region and political affiliation instead of school fixed effects, the results were very similar to our preferred model. It is important, however, that reading scores of Hispanic children were shown to increase with CEP participation even in the short term. The observed increase of a 0.055 standard deviation in the grade-adjusted reading z-scores is similar to the findings from other studies, including a 0.059 standard deviation increase for English Language Arts and 0.083 for math among non-poor students in NYC [18] and a 0.05 standard deviation increase in math in a national study [19]. Longer-term studies might be able to capture additional effects that improved access to nutritious school meals could bring to academic outcomes. 

Finally, our study did not identify significant changes in household food security after the CEP’s adoption, which would have been a major accomplishment of the UFM expansion. Relatively few families in the ECLS-K:2011 sample reported major disturbances in having continuous access to food, especially among children, so our statistical power could be limited. It is possible that the use of adult food security as a measure of household food insecurity was not sensitive enough to capture spillovers from children’s access to free school meals. There are no studies yet of the CEP effects on family finances [37] and future research should focus specifically on the effects of CEP expansion on food and nutrition security, including during and after the COVID-19 pandemic.

### Strengths and Limitations

This study is based on a nationally representative longitudinal sample of children with objective measures of key outcomes, including academic achievement and BMI, and survey data on school meal participation, attendance and household food security. To our knowledge, this is the first study that combines such a wide variety of outcomes in the CEP impact analysis. The longitudinal nature of the ECLK:2011 panel with geographic identifiers in its restricted-use version enable the use of a difference-in-difference model to estimate plausibly causal estimates of the CEP effects on a national sample of elementary schoolchildren. 

Along with its strengths, the study is subject to several important limitations. First, the study is limited to elementary schoolchildren who had the lowest non-participation rates among eligible students prior to CEP [9] and might benefit less from the CEP’s adoption than middle- or high schoolchildren. Failure to support the parallel trend assumption for key outcomes is another concern. Conducting a national study with large heterogeneity across states could explain some of our challenges in estimating the CEP effects as well as more modest estimated effects for some findings. The sample size in several sub-group analyses was small, which might have affected the results. Future research should study the effects of CEP and UFM, especially during and after COVID-19, on household food security and child nutrition. Our study did not have data on dietary effects of impacts of CEP expansion, which could be positive [5] and should be directly investigated in future research. Finally, studies of the CEP effects in older children are needed. 

## 5. Conclusions

This paper provides evidence that CEP participation in its early years increased school meal participation and attendance in elementary schools, without adverse effects on student academic achievement and body weight outcomes. Expanding access to UFM appears to particularly benefit at-risk children, including gains in reading scores for Hispanic children and reduced risk of being overweight among children from low-income families. The COVID-19 pandemic led to the UFM implementation across all schools, irrespective of student incomes [41], which could lead to broader adoption of CEP in the long term. Two states (CA and ME) have recently passed legislation to make UFM a permanent program [42,43]. Our work suggests that access to UFM, without disruptions of remote learning during the COVID-19 pandemic, will likely benefit children’s nutrition security and academic achievement, particularly among children from economically-vulnerable families. Future research should further investigate how UFM expansion can improve diet and reduce health and education disparities among American children. 

## Figures and Tables

**Table 1:** Survey-adjusted School Characteristics by CEP Participation and Eligibility Status in the Analytic Sample from the Early Childhood Longitudinal Study, Kindergarten Class of 2010–2011 (ECLS-K:2011).

School Characteristic	CEP Ineligible Schools	CEP Eligible, but Not Participating Schools	CEP Participating Schools
	Mean or % (95% CI)	Mean or % (95% CI)	Mean or % (95% CI)
% children eligible for free/reduced-price lunch	26.4 (24.6, 28.2)	57.2 (55.4, 59.0)	78.8 (76.8, 80.8) ***
% of poverty in school district	13.5 (12.9, 14.2)	20.1 (19.4, 20.7)	30.5 (29.4, 31.5) ***
Title I school	51.0 (46.7, 55.2)	80.1 (77.2, 83.1)	94.5 (92.3, 96.7) ***
Public school	76.5 (73.1, 79.8)	91.7 (89.7, 93.7)	98.4 (97.1, 99.7) ***
% of Hispanic children	19.5 (17.6, 21.3)	29.5 (27.5, 31.5)	26.8 (23.9, 29.6)
% of black non-Hispanic children	6.5 (5.5, 7.4)	13.1 (11.9, 14.4)	30.8 (28.0, 33.5) ***
% of white non-Hispanic children	59.6 (57.1, 62.1)	48.2 (46.0, 50.4)	26.6 (23.3, 30.0) ***
Suburban town	56.7 (53.0, 60.4)	55.5 (52.1, 58.9)	30.2 (25.7, 34.7) ***
Rural area	19.1 (15.9, 22.3)	23.3 (20.2, 26.5)	10.4 (7.3, 13.5) ***
Enrollment < 300 students	22.1 (17.8, 26.3)	18.0 (14.3, 21.7)	16.8 (12.0, 21.6)
Enrollment > 500 students	48.6 (43.9, 53.2)	51.9 (47.5, 56.2)	48.2 (42.2, 54.3)
Number of schools	1100	1400	700

CEP: Community Eligibility Provision; 95% CI: 95% confidence intervals. SOURCE: U.S. Department of Education, National Center for Education Statistics, Early Childhood Longitudinal Study, Kindergarten Class of 2010–2011 (ECLS-K:2011), 2011–2016. According to the regulations for restricted-use data produced by the Institute of Education Sciences Data Security Office, all sample sizes are rounded to the nearest 50. School level descriptive statistics were adjusted using a full sample school weight. *** *p* < 0.01.

**Table 2:** Child Characteristics by CEP Participation Status from the Early Childhood Longitudinal Study, Kindergarten Class of 2010–2011 (ECLS-K:2011).

Child Characteristic	Attending Non-CEP School	Attending CEP School
	Mean or % (95% CI)	Mean or % (95% CI)
White non-Hispanic	51.4 (51.1, 51.8)	24.5 (23.8, 25.2) ***
Black non-Hispanic	9.3 (9.1, 9.5)	23.4 (22.7, 24.0) ***
Hispanic	24.5 (24.2, 24.9)	38.2 (37.4, 39.0) ***
Asian	8.8 (8.6, 9.0)	9.0 (8.6, 9.5)
Other race	5.9 (5.7, 6.1)	4.9 (4.6, 5.3) ***
Male	51.2 (50.8, 51.5)	52.0 (51.2, 52.8) *
Primary language at home other than English	20.1 (19.8, 20.4)	33.0 (32.2, 33.8) ***
Child with disability	32.0 (31.6, 32.3)	32.8 (32.0, 33.6) *
Mother has less than high school education	11.2 (10.9, 11.5)	23.7 (22.8, 24.5) ***
Household income below 200% poverty threshold	33.5 (33.1, 33.8)	61.5 (60.7, 62.3) ***
% district poverty in child’s school	17.9 (17.8, 18.0)	29.2 (29.0, 29.4) ***
% of Hispanic students in child’s school	21.8 (21.6, 22.0)	34.7 (34.1, 35.3) ***
% of Asian students in child’s school	4.3 (4.3, 4.4)	3.8 (3.7, 3.9) ***
% of black non-Hispanic students in child’s school	10.7 (10.6, 10.8)	23.1 (22.7, 23.6) ***
% of white non-Hispanic students in child’s school	56.2 (55.9, 56.4)	28.6 (28.1, 29.1) ***
Child’s school receives funds from Title I	71.4 (71.0, 71.8)	94.3 (94.0, 94.7) ***
% of children in special education with an Individualized Education Program (IEP)	7.8 (7.8, 7.9)	8.8 (8.7, 8.9) ***
Attending public school	88.4 (88.2, 88.6)	98.3 (98.1, 98.5) ***
Attending suburban school	56.0 (55.6, 56.4)	34.4 (33.6, 35.2) ***
Attending rural school	26.3 (26.0, 26.6)	13.4 (12.8, 13.9) ***
Attending school with enrollment less than 300	16.9 (16.6, 17.2)	13.1 (12.5, 13.7) ***
Attending school with enrollment more than 500	54.9 (54.5, 55.3)	53.8 (52.9, 54.7) **
Reading proficiency z-score	0.066 (0.059, 0.074)	−0.318 (−0.334, −0.301) ***
Math proficiency z-score	0.069 (0.061, 0.076)	−0.329 (−0.346, −0.313) ***
Science proficiency z-score	0.075 (0.068, 0.082)	−0.367 (−0.383, −0.350) ***
% of students attending school daily in current school year	95.5 (95.5, 95.5)	95.0 (95.0, 95.1) ***
% of children reported by school administrators eligible for free or reduced-price lunch	43.4 (43.2, 43.6)	75.0 (74.5, 75.4) ***
% of parents reporting child’s receipt of free or reduced-price school lunch	44.2 (43.7, 44.7)	80.5 (79.6, 81.3) ***
BMI z-score	0.467 (0.456, 0.477)	0.606 (0.579, 0.633) ***
% overweight	15.7 (15.4, 15.9)	17.0 (16.4, 17.7) ***
% overweight/obese	32.0 (31.7, 32.4)	39.3 (38.5, 40.1) ***
Food secure household	91.9 (91.7, 92.2)	87.3 (86.6, 88.0) ***
Household with low food security	5.6 (5.4, 5.8)	9.2 (8.6, 9.9) ***
Household with very low food security	2.5 (2.3, 2.6)	3.5 (3.1, 3.9) ***
Number of children	15,300	2500

CEP: Community Eligibility Provision; 95% CI: 95% confidence intervals; BMI: body mass index. SOURCE: U.S. Department of Education, National Center for Education Statistics, Early Childhood Longitudinal Study, Kindergarten Class of 2010–2011 (ECLS-K:2011), 2011–2016. According to the regulations for restricted-use data produced by the Institute of Education Sciences Data Security Office, all sample sizes are rounded to the nearest 50. *** *p* < 0.01 ** *p* < 0.05 * *p* < 0.1.

**Table 3:** Estimated CEP Effects on Elementary Schoolchildren, 2011–2016.

		Sub-Groups of Elementary Schoolchildren			
Outcome	Full Sample β (95% CI) N	Low-Income β (95% CI) N	Hispanic β (95% CI) N	No Receipt of Free/Reduced Price School Meals before CEP β (95% CI) N	Primary Language at Home Other than English β (95% CI) N
	(1)	(2)	(3)	(4)	(5)
% of schoolchildren eligible for free lunch	**2.396** *** (1.493, 3.299) 12,750	**1.980** *** (0.894, 3.066) 7850	N.R.	1.288 (−0.609, 3.186) 2950	0.827 (−0.558, 2.211) 4400
Receipt of free lunch in parental report	**0.093** *** (0.065, 0.120) 7750	−0.007 (−0.031, 0.016) 4700	**0.041** ** (0.003, 0.078) 3100	**0.407** *** (0.347, 0.468) 1550	−0.014 (−0.050, 0.023) 2700
% students attending school	**0.244** *** (0.061, 0.426) 10,350	**0.301** ** (0.038, 0.565) 6300	N.R.	0.083 (−0.195, 0.361) 2400	**0.436** ** (0.077, 0.795) 3450
Reading proficiency z-score	N.R.	N.R.	**0.055** * (−0.008, 0.119) 5050	0.009 (−0.056, 0.073) 2950	0.053 (−0.011, 0.018) 4300
Math proficiency z-score	N.R.	N.R.	0.003 (−0.050, 0.056) 5050	N.R.	N.R.
Science proficiency z-score	N.R.	N.R.	−0.015 (−0.080, 0.051) 4950	−0.019 (−0.092, 0.054) 2950	−0.053 (−0.119, 0.013) 4200
BMI z-score	0.001 (−0.116, 0.117) 12,050	0.065 (−0.048, 0.179) 7400	−0.033 (−0.151, 0.085) 4800	−0.123 (−0.437, 0.190) 2800	0.012 (−0.110, 0.133) 4100
Overweight	−0.014 (−0.037, 0.009) 12,050	**−0.031** ** (−0.061, 0.000) 7400	−0.024 (−0.063, 0.016) 4800	0.003 (−0.037, 0.043) 2800	−0.027 (−0.067, 0.012) 4100
Obesity/overweight	0 (−0.020, 0.020) 12,050	−0.008 (−0.035, 0.018) 7400	0.008 (−0.027, 0.042) 4800	0.012 (−0.022, 0.045) 2800	0.002 (−0.034, 0.037) 4100
Food secure household	−0.013 (−0.035, 0.009) 8100	−0.022 (−0.058, 0.014) 4700	−0.009 (−0.052, 0.034) 3150	−0.012 (−0.035, 0.011) 1950	0.009 (−0.035, 0.053) 2750
Household with low food security	0.011 (−0.008, 0.031) 8100	0.022 (−0.010, 0.054) 4700	0.01 (−0.031, 0.051) 3150	0 (−0.016, 0.016) 1950	−0.002 (−0.042, 0.037) 2750
Household with very low food security	0.001 (−0.013, 0.014) 8100	−0.002 (−0.024, 0.021) 4700	−0.003 (−0.024, 0.018) 3150	N.R.	−0.008 (−0.033, 0.018) 2750

CEP: Community Eligibility Provision; 95% CI: 95% confidence intervals; BMI: body mass index. SOURCE: U.S. Department of Education, National Center for Education Statistics, Early Childhood Longitudinal Study, Kindergarten Class of 2010–2011 (ECLS-K:2011), 2011–2016. According to the regulations for restricted-use data produced by the Institute of Education Sciences Data Security Office, all sample sizes are rounded to the nearest 50. *** *p* < 0.01; ** *p* < 0.05; * *p* < 0.1. Statistically significant coefficients are bolded. All regressions include child random effects, school fixed effects, year fixed effects, and child-, and family-level covariates (child’s gender, race/ethnicity, household income below the 200% federal poverty level, primary language at home other than English, mother’s education, school-level time varying covariates (school district % of poverty, duration of CEP participation). BMI-based models also include child’s birth weight. N reports child-year observations.

## Data Availability

The ECLS-K data is available through the National Center for Education Statistics at the US Department of Education.

## Supplementary Materials

The following are available online at https://www.mdpi.com/article/10.3390/nu13082634/s1, Figure S1: Event Study, Results: Daily school attendance (%), Full Sample, Figure S2: Event Study, Results: School % of eligible for free or reduced-price lunch, Full Sample, Figure S3: Event Study, Results: Child’s receipt of complete school lunches for free or reduced price, Full Sample.
