# Supplementary material for: Universal School Meals in the US: What Can We Learn from the Community Eligibility Provision?
Source: Nutrients. 2021 Jul 30;13(8):2634. doi: 10.3390/nu13082634 (PMC8398513; doi:10.3390/nu13082634)
Supplement: Supplementary file 1 [file nutrients-13-02634-s001.zip › nutrients-1304134-supplementary.pdf]

Figure S1. Event Study, Results: Daily school attendance (%), Full Sample

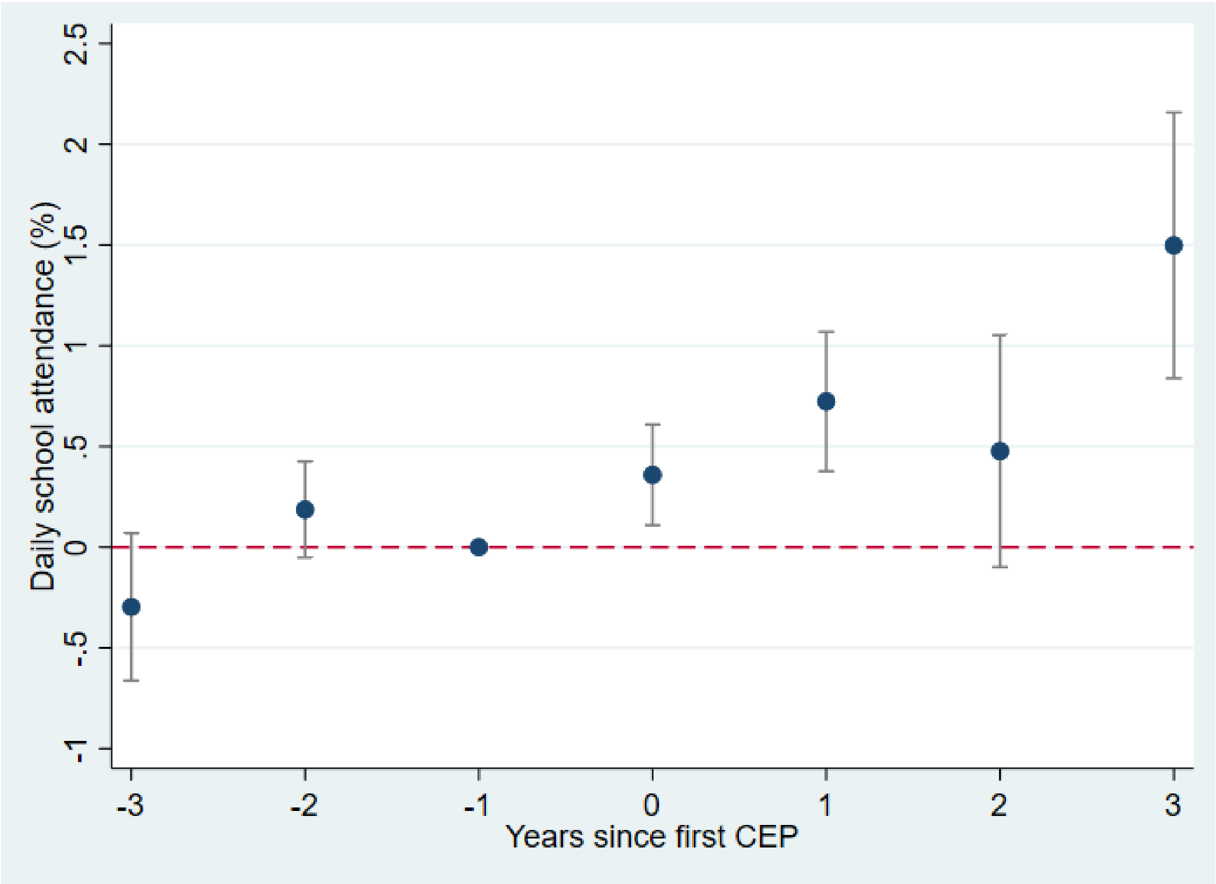

SOURCE: U.S. Department of Education, National Center for Education Statistics, Early Childhood Longitudinal Study, Kindergarten Class of 2010-11 (ECLS-K:2011), 2011-2016.
Notes: CEP: Community Eligibility Provision.
Figures display point estimates and corresponding 95 percent confidence intervals for the coefficients in event study.
The year just prior to first CEP exposure (-1) is the reference year.
All regressions include child random effects, school fixed effects, year fixed effects, and child-, and family-level covariates such as child's gender, race/ethnicity, household income below the 200% federal poverty level, primary language at home other than English and mother's education, and school-level time varying covariates such as percentage of poverty in a school district and the duration of the CEP participation by the school.

Figure S2. Event Study, Results: School % of eligible for free or reduced-price lunch, Full Sample

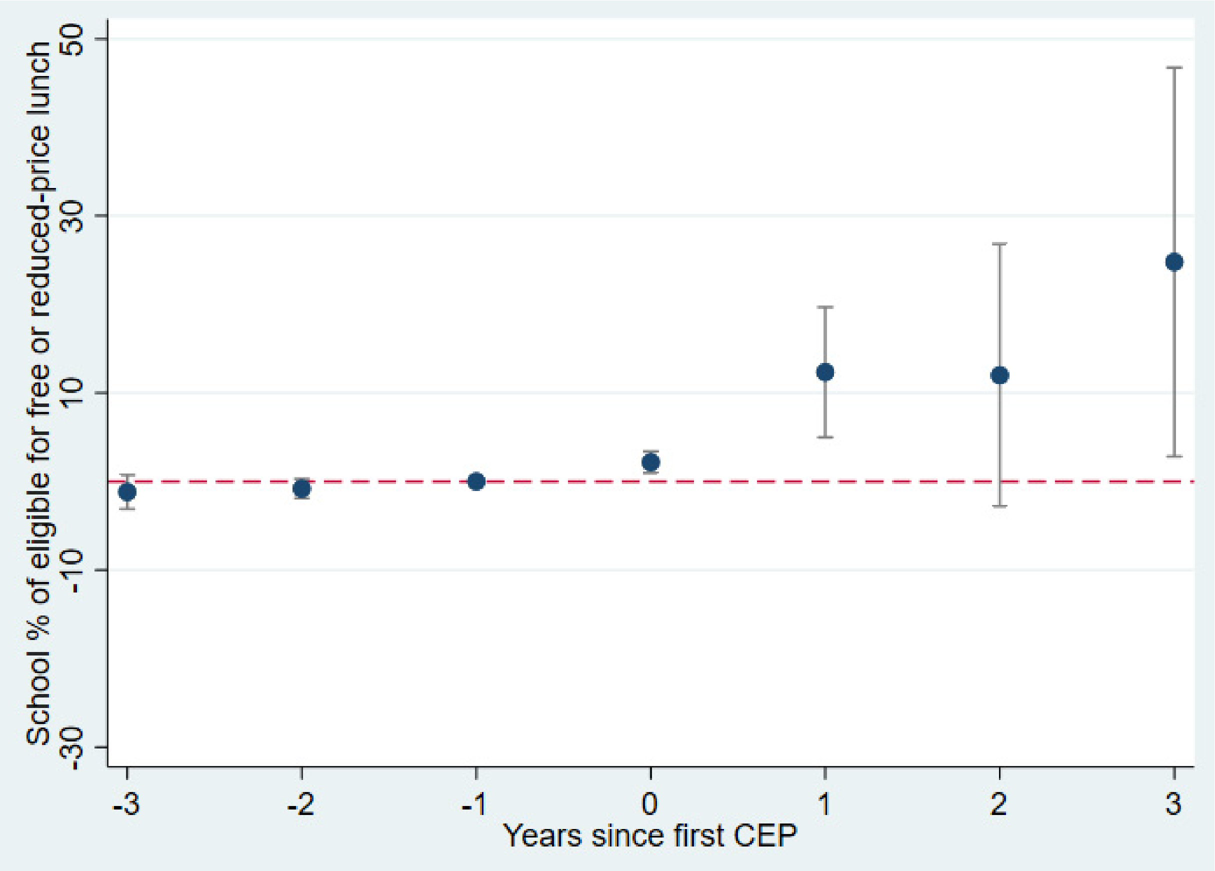

SOURCE: U.S. Department of Education, National Center for Education Statistics, Early Childhood Longitudinal Study, Kindergarten Class of 2010-11 (ECLS-K:2011), 2011-2016.
Notes: CEP: Community Eligibility Provision.
Figures display point estimates and corresponding 95 percent confidence intervals for the coefficients in event study.
The year just prior to first CEP exposure (-1) is the reference year.
All regressions include child random effects, school fixed effects, year fixed effects, and child-, and family-level covariates such as child's gender, race/ethnicity, household income below the 200% federal poverty level, primary language at home other than English and mother's education, and school-level time varying covariates such as percentage of poverty in a school district and the duration of the CEP participation by the school.

Figure S3. Event Study, Results: Child's receipt of complete school lunches for free or reduced price, Full Sample

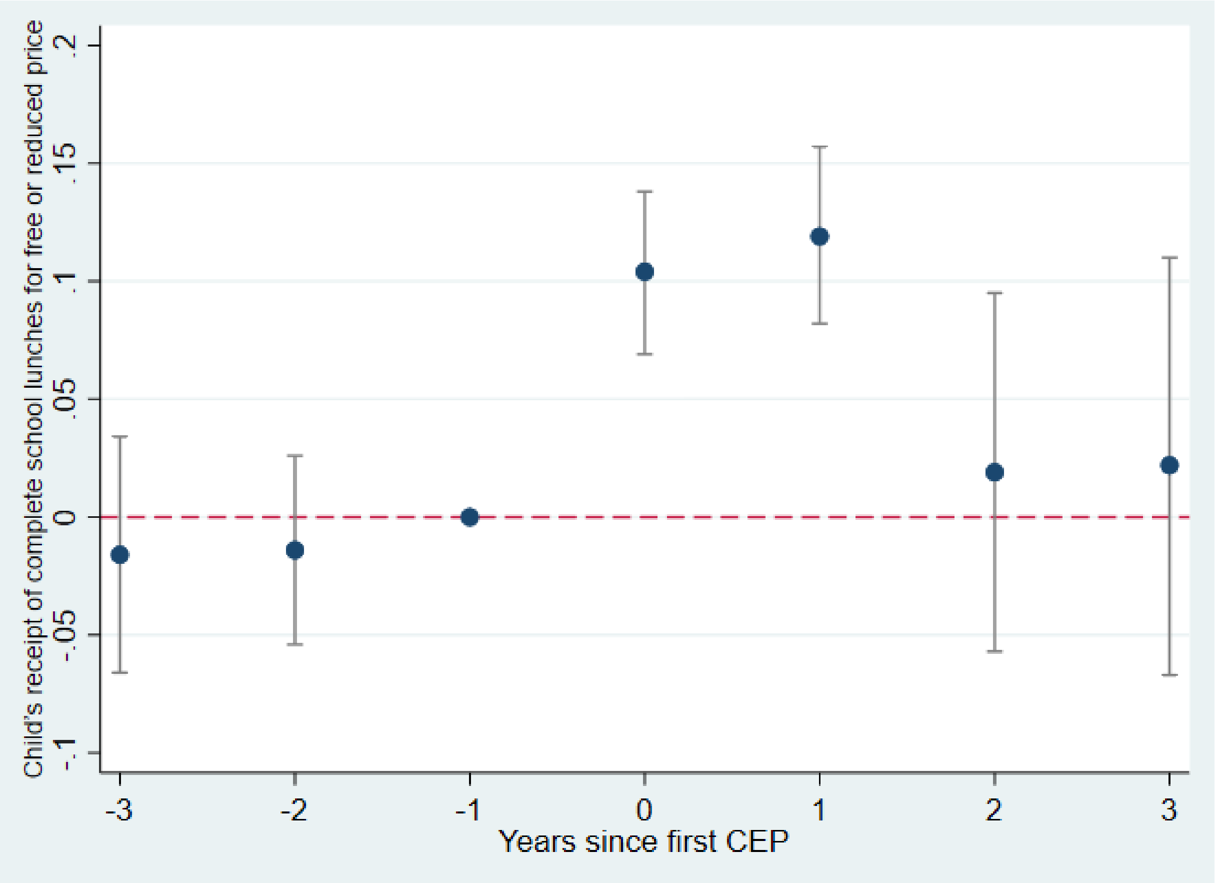

SOURCE: U.S. Department of Education, National Center for Education Statistics, Early Childhood Longitudinal Study, Kindergarten Class of 2010-11 (ECLS-K:2011), 2011-2016.
Notes: CEP: Community Eligibility Provision.
Figures display point estimates and corresponding 95 percent confidence intervals for the coefficients in event study.
The year just prior to first CEP exposure (-1) is the reference year.
All regressions include child random effects, school fixed effects, year fixed effects, and child-, and family-level covariates such as child's gender, race/ethnicity, household income below the 200% federal poverty level, primary language at home other than English and mother's education, and school-level time varying covariates such as percentage of poverty in a school district and the duration of the CEP participation by the school.
